# Histological Subtypes Drive Distinct Prognostic Immune Signatures in Classical Hodgkin Lymphoma

**DOI:** 10.3390/cancers14194893

**Published:** 2022-10-06

**Authors:** Claire Lamaison, Juliette Ferrant, Pauline Gravelle, Alexandra Traverse-Glehen, Hervé Ghesquières, Marie Tosolini, Cédric Rossi, Loic Ysebaert, Pierre Brousset, Camille Laurent, Charlotte Syrykh

**Affiliations:** 1 Centre Hospitalier Universitaire de Rennes, Pôle Biologie, 35033 Rennes, France; 2 Institut National de la Santé et de la Recherche Médicale, INSERM, Unité Mixte de Recherche U1236, Université Rennes 1, Établissement Français du Sang Bretagne, 35000 Rennes, France; 3 Service d’Anatomopathologie, Centre Hospitalier Universitaire de Toulouse, Institut Universitaire du Cancer de Toulouse-Oncopole, 31059 Toulouse, France; 4 Centre de Recherches en Cancérologie de Toulouse, INSERM, U1037, 31037 Toulouse, France; 5 Laboratoire d’Excellence TOUCAN, 31037 Toulouse, France; 6 Service d’Anatomopathologie, Centre Hospitalier Universitaire de Lyon-Sud, 69495 Lyon, France; 7 Service d’Hématologie, Centre Hospitalier Universitaire de Lyon-Sud, Université Claude Bernard Lyon-1, Pierre-Bénite, 69495 Lyon, France; 8 Service d’Hématologie, Centre Hospitalier Universitaire Dijon, Hôpital François Mitterrand, 21000 Dijon, France; 9 Service d’Hématologie, Centre Hospitalier Universitaire de Toulouse, Institut Universitaire du Cancer de Toulouse-Oncopole, 31059 Toulouse, France; 10 Université de Toulouse III Paul Sabatier, 31062 Toulouse, France

**Keywords:** Hodgkin lymphoma, immune prognosis signature, histological subtypes, gene expression profiling

## Abstract

**Simple Summary:**

Classical Hodgkin lymphoma (cHL) is a highly curable disease, with about 80% of patients cured using standard first-line chemotherapy. However, outcomes for relapsed/refractory patients remain unfavorable and there is a critical lack of predictive biomarkers for early identification of these patients who may benefit from new therapeutic strategies. Here we evaluated the dynamic expression of 586 immune-related genes in a cohort of 42 cHL patients using NanoString technology. We identified a 19-gene immune signature predictive of relapse at the time of diagnosis, which was found to be strongly dependent on histological subtype. Moreover, comparative analyses between paired diagnostic/relapsed biopsies of nodular sclerosis cHL showed 118 differentially expressed genes, highlighting an immune contexture shift at relapse not found in mixed-cellularity cases. Overall, these results strongly suggest that the predictive value of immune signature in cHL is influenced by histological subtype, a criterion that should be considered when assessing new immunotherapy strategies.

**Abstract:**

Despite the success of standard front-line chemotherapy, 20% of classical Hodgkin lymphoma (cHL) patients still relapse or have refractory disease (r/r), and a subset of them die due to disease progression. There is a critical lack of predictive factors for early identification of those r/r patients who may benefit from new therapeutic strategies. This study aimed to evaluate the dynamic expression of 586 immune-related genes in a cohort of 42 cHL patients including 30 r/r cHL after first-line chemotherapy. Gene expression profiling (GEP) using NanoString technology identified a 19-gene immune signature at diagnosis predictive of cHL relapse, but dependent on histological subtypes. Genes related to tumor survival were found upregulated while genes related to B-lineage were downregulated at diagnosis in r/r nodular sclerosis cHL. In contrast to the mixed-cellularity subtype, comparative GEP analyses between paired diagnosis/relapse biopsies of nodular sclerosis cHL showed 118 differentially expressed genes, supporting an immune contexture switch at relapse with upregulation of immunosuppressive cytokines, such as *LGALS1* and *TGFB1*, and downregulation of the T-cell co-stimulatory receptor *ICOS*. These results indicate that the predictive value of immune signature in cHL is strongly influenced by histological subtype which should be considered when assessing new immunotherapy target strategies.

## 1. Introduction

Classical Hodgkin lymphoma (cHL) is a lymphoid neoplasm characterized by a paucity of neoplastic cells, embedded in a reactive cellular infiltrate of variable composition. cHL is divided into four histological subtypes according to the number and morphological characteristics of the Hodgkin Reed-Sternberg (HRS) cells as well as the composition of the reactive infiltrate, including: nodular sclerosis (70% of cases), mixed-cellularity (15–25%), lymphocyte-rich (5%) and lymphocyte depletion (1%) [1,2,3]. Prognosis depends on several factors, notably disease stage and other associated co-morbid illnesses. In contrast to earlier reports, recent research does not regard histological subtype as a major prognostic indicator [4,5]. Despite conflicting results, gene signatures and immunohistochemistry (IHC) analyses of tumor microenvironment (TME) have been shown to be associated with distinct outcomes in cHL patients [6,7,8,9,10,11,12,13,14,15,16]. In addition, Sanchez et al. described specific gene signatures associated with treatment response in patients with cHL, involving genes that contribute to mitosis regulation and cell growth/apoptosis as well as genes related to host immune response and TME [16]. More recently, Jachimowicz et al. advanced the potential of gene expression profiling (GEP) approaches for pre-treatment risk assessment of cHL patients [15].

cHL is generally considered to be a highly curable disease, with approximately 80% of patients cured with standard first-line chemotherapy. However, 20% of cHL patients still relapse or have a refractory disease to first line therapy (r/r cHL), and a subset of these patients die despite aggressive chemotherapy regimens and stem cell transplantation [17,18]. Recently, targeted therapy, such as anti-CD30 brentuximab vedotin and immune checkpoint inhibitors (antibodies against CTLA-4 or PD-1/PD-L1), have been approved for the treatment of r/r cHL, in which response rates to PD-1 inhibitors nivolumab or pembrolizumab exceed 60% [19]. Despite such treatments, complete remissions and durable responses remain uncommon in r/r cHL, and predictive factors are lacking for accurately identifying r/r patients that could benefit from new therapeutic strategies.

In our study, we aimed to identify immune-related gene expression in r/r cHL patients by using the NanoString nCounter Human Immunology Panel V2. We first investigated whether immune gene signatures could predict disease relapse or progression in cHL patients at initial diagnosis. Then, we analyzed the immune GEP and its prognostic value according to cHL histological subtypes. Finally, we compared immune GEP in paired biopsies at diagnosis and relapse to identify hallmark immune genes associated with r/r cHL, according to histological subtypes.

## 2. Materials and Methods

### 2.1. Samples

Sixty-six formalin fixed paraffin-embedded (FFPE) cHL biopsies at diagnosis and/or at relapse were obtained from a total of 42 patients, whose characteristics are shown in Table 1. Median tumor cell content of FPPE blocks was 6% (range 1–15%). Patients were recruited under institutional review board approval upon an in-formed consent process according to the declaration of Helsinki and the French National Cancer Institute ethics committee recommendations. RNA from each sample was extracted using the High Pure FFPET RNA Isolation Kit (Roche), following the manufacturer’s protocol. For each RNA extraction, the tumor content was confirmed through histopathological evaluation performed by a certified pathologist. All 66 biological samples passed quantification and quality controls (QC) using the Agilent Bioanalyzer (BA) (2100 Bioanalyzer Instrument, Santa Clara, CA, USA).

### 2.2. NanoString Protocol: Sample Preparation and Hybridization

All samples were analyzed using the nCounter Human Immunology V2 panel with Panel Plus (comprising 579 immune-related genes, 7 custom genes, and 15 housekeeping genes, Supplementary Table S1) in accordance with the manufacturer’s guidelines. In brief, 5 μL of each sample were mixed with 8 μL of the hybridization cocktail (4 μL of the reporter codeset and 4 μL of hybridization buffer). Two μL of the capture codeset were added; the solution was mixed and spun down. It was then hybridized in a 65 °C thermocycler (Veriti Thermal Cycler, Applied Biosystems, Foster City, CA, USA) for 16 h. Hybridized samples were analyzed on the nCounter NanoString Preparation Station using the high-sensitivity protocol, whereby excess capture and reporter probes were washed away and ternary target probe complexes were immobilized on a streptavidin-coated cartridge. Samples were scanned at maximum resolution on the nCounter Digital Analyzer (NanoString Technologies, Seattle, WA, USA).

### 2.3. Data Processing and Analysis

Data were analyzed using ROSALIND^TM^ (https://rosalind.onramp.bio/), with a HyperScale architecture developed by ROSALIND, Inc. (San Diego, CA, USA). Read Distribution percentages, identity heatmaps, and sample Multidimensional Scaling (MDS) plots were generated as part of the QC step. Housekeeping probes used for normalization were selected based on the geNorm algorithm, as implemented in the NormqPCR R library. Fold changes and *p*-values were calculated using nCounter® Advanced Analysis 2.0 User Manual. P-value adjustment was performed using the Benjamini-Hochberg method of estimating false discovery rates (FDR). Hypergeometric distribution was used to analyze the enrichment of pathways. The topGO R library was used to determine local similarities and dependencies between GO terms in order to perform Elim pruning correction. Several database sources were referenced for enrichment analysis, including Reactome. Further analyses were performed and figures were generated with GraphPad Prism 9.0.0 and R v3.6.3, using RStudio v1.2.5033. Hierarchical clustering and heatmaps were generated with the pheatmap package. Volcano plots were obtained using the EnhancedVolcano package. Lastly, the correlation matrix was created with the corrplot package.

## 3. Results

### 3.1. Clinical and Histological Features of Patients Included in the Study

Forty-two cHL patients were enrolled in the study, including 30 r/r cHL patients and 12 non-r/r cHL patients. None of them had personal history of autoimmune disease or associated lymphoma. Their clinical and histological characteristics are reported in Table 1. Thirty-five FFPE samples were available at diagnosis (n = 12 non-r/r cHL and n = 23 r/r cHL) and FFPE paired diagnosis and relapse biopsies were available for 22/30 r/r cHL patients. Nodular sclerosis subtype was the most represented histological subtype in our series (n = 24/42, 57.1%) followed by the mixed-cellularity subtype (n = 15/42, 35.7%). Only two patients presented a lymphocyte-rich pattern (4.8%), and one patient displayed a lymphocyte-depleted pattern (2.4%). The average age at diagnosis of r/r patients was 30 years (ranging from 15y to 83y) and that of non-r/r patients was 31 years (ranging from 23y to 58y). Less than 25% (n = 9/39) of cases were Epstein-Barr virus (EBV) positive and were equally distributed between nodular sclerosis and mixed-cellularity subtypes (20% and 21% of cases, respectively). EBV status was unknown for three cases. Thirty-two (78%) patients, including 20/30 r/r patients and 12/12 non-r/r patients, had extensive disease at diagnosis (Ann Arbor stage III–IV). Of the 40 assessable patients, 30.9% (n = 13) were treated with bleomycin, etoposide, doxorubicin, cyclophosphamide, vincristine, procarbazine and prednisone (BEACOPP) and 57.1% (n = 24) received doxorubicin, bleomycin, vinblastine, and dacarbazine (ABVD). The 3 remaining patients received alternative chemotherapy (Table 1). None of the patients had received immune checkpoint inhibitors and treatment types were homogeneous among the histological subtypes. All patients who did not relapse achieved a complete response after first-line therapy. Among r/r patients, 23 had initial complete response and 6 experienced disease progression requiring therapeutic change. The median time between initial cHL diagnosis and relapse was 19 months (ranging from 4 months to 10 years). After a median follow-up of 43 months (ranging from 5 months to 10 years), 34/42 patients (including 22 r/r patients) were alive, 7/30 r/r cHL patients had died of lymphoma progression within 3–46 months after the relapse and one patient had died of a second malignancy.

### 3.2. Gene Expression Profiling Identifies an Immune Signature Predictive of cHL Relapse

**Figure 1:**
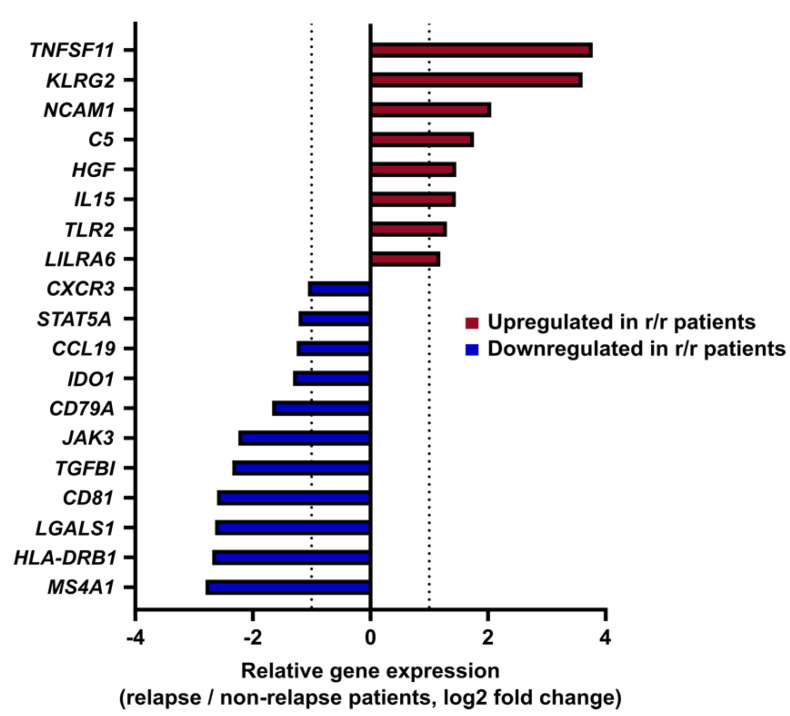
Prognostic immune gene signature in classical Hodgkin lymphoma: Differentially expressed genes (*p*-value < 0.05 and |fold change| > 2) in relapse patients compared to non-relapse patients at diagnosis, regardless of the histological subtype.

To compare immune profiles between r/r and non-r/r patients at initial diagnosis, GEP using the Human Immunology V2 NanoString panel of 586 immune genes was first evaluated by unsupervised analysis with a MDS approach, to explore dissimilarity between the two groups after normalization and log2 count transformation. As shown in Supplementary Figure S1, cHL samples were inconsistently distributed and showed no clustering according to disease outcome, suggesting that unsupervised analysis of immune-related gene profiles in cHL at diagnosis was more driven by inter-patient heterogeneity than disease progression. We thus focused our analysis on differential immune gene expression at initial diagnosis between r/r and non-r/r patients. Global differential gene expression analysis from all cHL samples at diagnosis revealed 8 upregulated genes (*TNFSF11, KLRG2, NCAM1, C5, HGF, IL15, TLR2, LILRA6*) and 11 downregulated genes (*CXCR3, STAT5A, CCL19, IDO1, CD79A, JAK3, TGFBI, CD81, LGALS1, HLA-DRB1, MS4A1*) in r/r patients as compared to non-r/r patients (*p*  <  0.05) with a consistent |FC| > 2 (Figure 1).

### 3.3. The Immune Prognostic Signature Depends on the cHL Histological Subtype

A major parameter of cHL tumor heterogeneity is histological subtype. Consistent with the literature, nodular sclerosis and mixed-cellularity subtypes were the most represented, in our cohort. As shown in Supplementary Figure S2, these 2 subtypes exhibited distinct GEP at diagnosis, including 30 genes significantly upregulated in the mixed-cellularity subtype and 4 genes upregulated in the nodular sclerosis subtype, all outcomes combined, showing immune niche divergences between these two entities. To assess the impact of histological subtypes on the immune prognostic signature, we compared the immune GEP at initial diagnosis of r/r cHL patients versus non-r/r patients for nodular sclerosis and mixed-cellularity cHL samples, separately.

**Figure 2:**
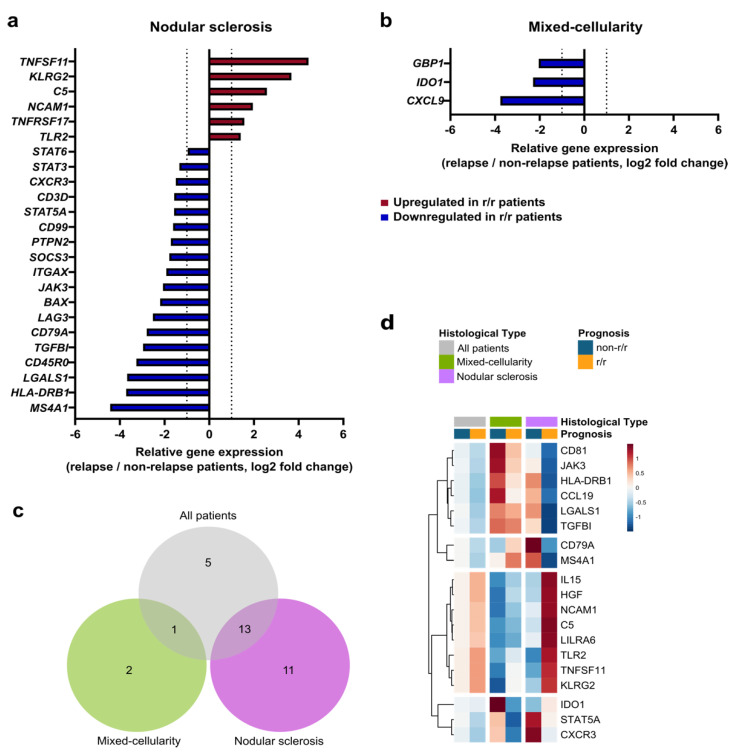
Immune-related gene prognostic signature according to classical Hodgkin lymphoma histological subtypes: (**a**) and (**b**) differentially expressed genes (*p*-value < 0.05 and |fold change| > 2) at diagnosis in r/r compared to non-r/r patients in nodular sclerosis and mixed-cellularity subtypes, respectively; (**c**) Venn diagram showing the intersection of differentially expressed genes at diagnosis (*p*-value < 0.05 and | fold change| > 2) between r/r and non-r/r patients for all cHL cases and according to the subtypes; (**d**) heatmap showing the average expression of the prognostic genes identified in Figure 1 according to r/r and non-r/r status, for all patients and for mixed-cellularity and nodular sclerosis sub-cohorts (z-score normalized counts mean).

At initial diagnosis in the nodular sclerosis subtype, we identified 6 upregulated genes (*TNFSF11, KLRG2, C5, NCAM1, TNFRSF17, TLR2*), and 18 downregulated genes (*STAT6, STAT3, CXCR3, CD3D, STAT5A, CD99, PTPN2, SOCS3, ITGAX, JAK3, BAX, LAG3, CD79A, TGFBI, CD45RO, LGALS1, HLA-DRB1, MS4A1*) (*p* < 0.05) in r/r patients as compared to non-r/r patients (Figure 2a). By contrast, the mixed-cellularity subtype showed only three differentially expressed genes between r/r and non-r/r patients (Figure 2b). These three genes (*GBP1, IDO1*, *CXCL9*) were all downregulated in r/r patients (*p* < 0.05). As summarized in Figure 2c, 13 out of the 19 differentially expressed genes identified in the global immune prognostic signature were also identified in the nodular sclerosis pattern signature, but only one was found in the mixed-cellularity signature. Five genes of the global immune prognostic signature did not show significant differential expression according to the subtype. Interestingly, we noticed that nodular sclerosis and mixed-cellularity cHL did not share any differentially expressed genes related to relapse. The heatmap of the global cHL immune prognosis signature showed that the nodular sclerosis subtype exhibited the most distinctive GEP at initial diagnosis between r/r and non-r/r patients (Figure 2d). By contrast, this global signature did not appear to discriminate r/r from non-r/r patients with a mixed-cellularity subtype. Additionally, we wondered if this gene expression signature may be influenced by disease stages and performed a correlation analysis that found no relevant correlations between gene expression levels and the Ann Arbor stage (Supplementary Figure S3).

### 3.4. Comparative Analysis of Paired Diagnosis and Relapse Samples Reveals Distinct Immune Profiles

**Figure 3:**
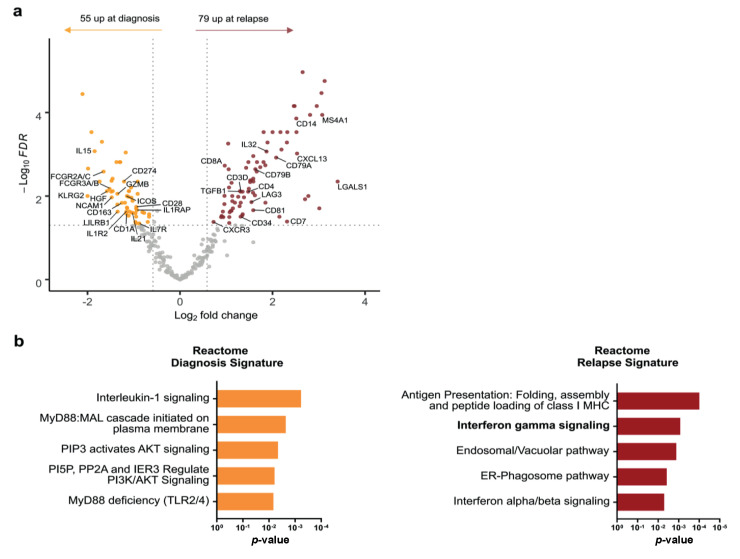
Differential gene expression analysis in paired classical Hodgkin lymphoma samples at diagnosis and relapse: (**a**) volcano plot displaying differentially expressed genes between samples at diagnosis and at relapse. Significantly de-regulated genes (FDR < 0.05 and |fold change| > 1.5) are highlighted in orange (upregulated at diagnosis, n = 55) or red (upregulated at relapse, n = 79); (**b**) top 5 increases in biological processes at diagnosis (orange, left) and at relapse (red, right) identified with REACTOME database, ranked by *p*-value. FDR = False Discovery Rate.

To explore immune profile evolution before and after treatment in r/r patients, 22 r/r patients (including 12 nodular sclerosis, eight mixed-cellularity, and two lymphocyte-rich subtypes) with available paired samples at diagnosis and at relapse were selected for comparison of their immune GEP using pairwise analysis. All cases but one in our cohort presented the same histological subtype at both initial diagnosis and relapse. One patient presented a nodular sclerosis pattern at diagnosis but a mixed-cellularity pattern at relapse. We observed gene expression variations in 134 immune-related genes between diagnosis and relapse, reflecting the dynamics of the immune TME (Figure 3a and Supplementary Table S2). Fifty-five genes were significantly upregulated at diagnosis compared to paired relapse samples (FDR < 0.05, |FC| > 1.5), including *CD274*, *ICOS*, *KLRG2*, and *CD163*, which are mainly involved in immune escape mechanisms, and T-cell activation genes such as *GRZB*, *IL15,* and *IL1R2*. Conversely, 79 genes were significantly upregulated at relapse compared to the paired diagnosis samples (FDR < 0.05, |FC| > 1.5) including *CD81*, *LAG3*, *TGFB1*, and *IL32*. Based on the REACTOME database, the upregulated genes at diagnosis were involved in innate immune response pathways, especially in the interleukin-1 (IL-1) signalling and MYD88 pathways (Figure 3b). By contrast, relapse cHL samples showed a distinct immune contexture with upregulation of genes involved in antigen presentation and type I and II interferon signalling pathways, regulating both innate and adaptive immune responses (Figure 3b).

### 3.5. Changes in cHL Immune Environment at Relapse Depend on Histological Subtype

**Figure 4:**
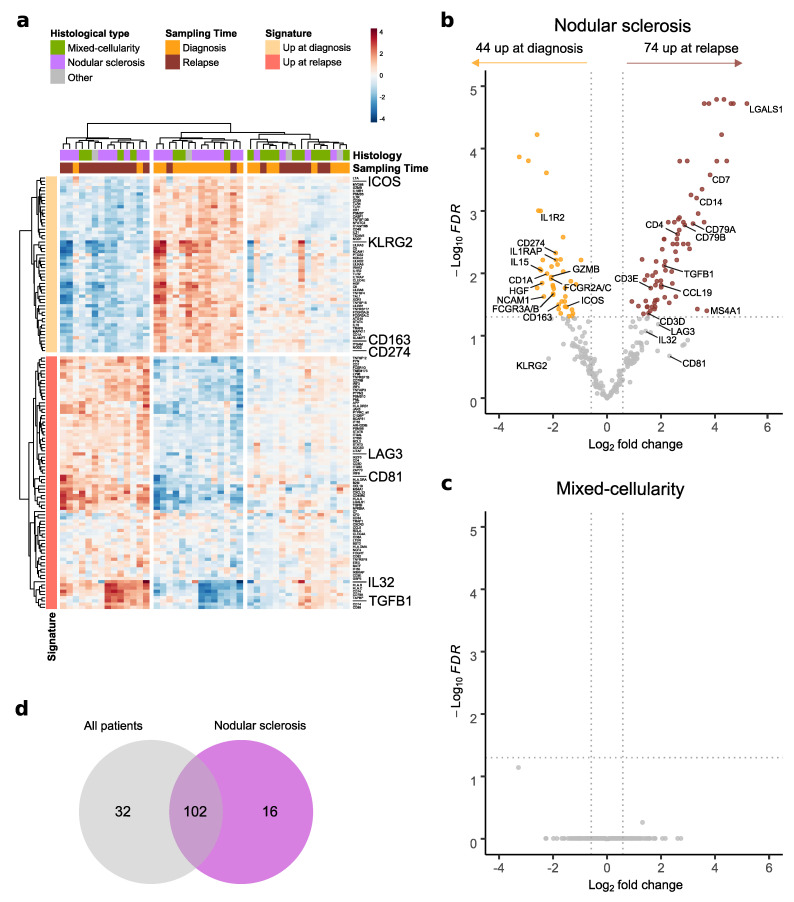
Differential gene expression analysis in paired diagnostic and relapse samples according to histological subtypes of classical Hodgkin lymphoma: (**a**) heatmap of the significantly deregulated genes between relapse and diagnosis (see Figure 3a and Supplementary Table S2) across all paired samples, annotated by sampling timepoint and histological subtype (log2 fold change); (**b**) and (**c**) volcano plot displaying differentially expressed genes (FDR < 0.05 and |fold change| > 1.5) between diagnostic and relapse samples in the nodular sclerosis subtype and in the mixed-cellularity subtype, respectively; (**d**) Venn diagram displaying the intersection of differentially expressed genes (FDR < 0.05 and |fold change| > 1.5) between relapse and diagnosis from all paired samples and nodular sclerosis sub-cohorts. FDR = False Discovery Rate.

As histological patterns appeared to determine distinct immune-prognostic profiles (Figure 2), we investigated whether histological subtypes could also influence the immune profile evolution in cHL tissue from diagnosis to relapse. For this part of the analysis, only patients with nodular sclerosis or mixed-cellularity subtypes were sufficiently represented, and thus included (n = 20). As shown in Figure 4a, the immune-related gene signature based on the 134 differentially expressed genes (Figure 3a and Supplementary Table S2) segregated the nodular sclerosis samples according to biopsy timepoint, but not the mixed-cellularity samples. Next, we performed independent analyses of differentially expressed genes at relapse versus diagnosis on the 16 matched biopsies with a mixed-cellularity pattern and on the 22 matched biopsies with a nodular sclerosis pattern. In the group of patients presenting a nodular sclerosis subtype, the immune profile switched at relapse (Figure 4b and Supplementary Table S3), with an upregulation of *LGALS1* and *TGFB1* and downregulation of *CD163, CD274, HGF, IL15*, and *ICOS*. However, no immune-related genes were significantly differentially expressed between diagnosis and relapse in the mixed-cellularity subtype group (Figure 4c). Consistent with this observation, a correlation matrix of the mixed-cellularity samples reveals strong inter- and intra-patient correlation with similar immune GEP between diagnosis- and relapse-matched samples (Supplementary Figure S4). Among the 118 genes found differentially expressed between diagnosis and relapse in nodular sclerosis cHL samples (Supplementary Table S3), 102 overlapped with the global signature (Figure 4d).

## 4. Discussion

In this study, we identified an immune prognostic signature in cHL based on the differential expression of 19 genes involved in immune responses, using NanoString nCounter technology. We further showed that this immune signature was linked to the histological subtype of cHL and, depending on the subtype, could identify a subset of cHL patients at high risk of relapse.

Genes downregulated at diagnosis in r/r cHL patients compared to non-r/r patients were mostly related to B-cell differentiation or activation. Among them, *CD79A, MS4A1, CD74, CD81* genes have already been identified as favorable prognosis factors in cHL patients [20,21,22,23,24]. Indeed, it has been demonstrated that loss of B-lineage identity allows HRS cells to survive in the absence of BCR signaling, and prevents the induction of apoptosis [25,26]. Notwithstanding that the therapeutic potential of restoring the lost B-cell phenotype of HRS cells was recently proposed by Du et al. [27], the prognostic impact of B-cell markers expression by HRS cells remains controversial [28,29,30]. In addition, some genes involved in cell proliferation such as *TNFSF11 (RANKL), HGF*, and *IL15* genes were upregulated at diagnosis in r/r patients. Interestingly, these cytokines and their corresponding receptors are known to be expressed by cHL cells and promote tumor growth through autocrine signaling [31,32,33]. By contrast, the gene coding for the CXCR3 chemokine receptor was found downregulated in r/r patients at diagnosis compared to non-r/r patients. *CXCR3* is known to be expressed by both Th1 CD4^+^ T-cells and CD8^+^ effector T-cells and plays an important role in T-cell homing to the tumor site [34]. Such *CXCR3* downregulation may reflect impaired Th1-polarized immune response in r/r patients, well-known for its central role in tumor cell eradication and previously described in cHL [35].

By applying our immune signature to the nodular sclerosis and mixed-cellularity subtypes separately, we showed that its prognostic value varies between these two cHL subtypes that are characterized by distinct TME, including various immune and stromal cell subsets as well as extracellular matrix components. Nodular sclerosis cHL is constituted by inflammatory nodules with admixed HRS cells, surrounded by collagen bands. In contrast, the mixed-cellularity pattern lacks the nodular sclerosis but contains a more abundant and polymorphic infiltrate, sometimes with clusters of epithelioid histiocytes [2]. In our study, genes related to major histocompatibility complex (MHC) class I and II machinery/biosynthesis such as *HLA-DRA*, *CTSS*, *HLA-DPA1*, *HLA-DPB1*, *CTSC*, *B2M* genes were expressed at higher levels in the mixed-cellularity subtype than in nodular sclerosis subtype at diagnosis. However, despite a strong immune TME in mixed-cellularity cHL, we did not identify a robust immune prognostic signature in this subtype suggesting that different mechanisms of tumor progression may be involved in this subtype. One could argue that the dedicated panel, though based on 586 immune related genes, was not comprehensive enough to explore all immune evasion mechanisms involved in the mixed-cellularity subtype or that this subtype was under represented in our cohort compared to the nodular sclerosis (representing 35.7% and 57.1% of the whole cohort, respectively). We could further hypothesize that prognostic factors in the mixed-cellularity subtype may also include HRS cell-specific genes. Indeed, previous sequencing and gene expression studies suggest that the genotype and phenotype of HRS cells both strongly influence cellular crosstalk with TME [6,23,36]. Genetic alterations also differ substantially in frequency across histological subtypes and may influence immune cell behavior. For instance, inactivating mutations of the beta-2-microglobulin gene (*B2M*), which are mostly associated with the nodular sclerosis variant [36], lead to MHC class I molecule expression loss and thus impair the ability of immune effector cells to recognize and interact with cells harboring those mutations [37]. Finally, other parameters may affect the immune microenvironment but due to the limited number of cases, we did not perform multivariate analyses (including, for example, EBV status, age, disease stage, and treatment) that would be worth performing on an additional larger cohort. Overall, these observations underline the crucial need to consider the histological subtype of cHL when evaluating potential prognostic or predictive biomarkers.

Beyond prognostic signatures at diagnosis, we also sought to investigate immune TME modifications between diagnosis and relapse in paired samples. Our results showed a wide range of changes in the immune contexture in nodular sclerosis subtype throughout disease progression, whereas no genes were found to be differentially expressed between diagnosis and relapse in mixed-cellularity cHL. Comparison between paired diagnostic and relapse samples in the nodular sclerosis subtype showed upregulation of immunosuppressive cytokines such as *LGALS1* (encoding for galectin-1) and *TGFB1* (encoding for TGF-β) and downregulation of the T-cell costimulatory receptor *ICOS* at relapse, reflecting some measure of immune-evasion. TGF-β is thought to be responsible for the formation of fibrous bands in the nodular sclerosis subtype [38]. In this respect, TGF-β upregulation in nodular sclerosis relapse biopsies highlights the strong effect of the deregulation of cytokine-mediated immune response in disease progression. Conversely, other immunoregulatory genes, such as *CD274* (coding for PD-L1), *IL1R2* (decoy IL1 receptor), and *CD163* (marker of M2 and tumor-associated macrophages) were found to be upregulated at the time of diagnosis. Programmed cell death protein 1 (PD-1)/PD-L1 signaling contributes to promote cHL growth by allowing PD-L1+ HRS cells to escape the host immune system. In this respect, immune checkpoint inhibitors blocking the PD-1/PD-L1 axis have demonstrated remarkable efficacy in r/r patients [39] and are now being assessed as a component of frontline therapy with encouraging results [40,41]. In this setting, it has been shown that PD-L1+ tumor-associated macrophages were predominantly affected early after the start of anti-PD-1 first-line treatment [42]. In contrast, a recent study based on 13 paired biopsies did not show any changes in terms of PD-L1 and CD163 expression between relapse and initial diagnosis [21]. This discrepancy could be explained by the fact that, in the latter study, PD-L1 expression was not assessed according to histological subtypes. Besides, the decoy IL-1 receptor IL-1R2 was also found highly expressed in HRS cells and may contribute to tumor-induced immune escape mechanisms by inhibition of IL-1 signal transduction [43]. Finally, the expression of *IL-15* and *HGF* genes, known to promote tumor cell survival [31,44], was higher at diagnosis than in paired relapse samples. Those unexpected results may suggest that upon relapse, nodular sclerosis cHL progression might rely on TME changes more than on autocrine regulation of tumoral cells. Indeed, the distinct immunosuppressive environment found between diagnostic and relapse biopsies suggests that cHL exhibits different mechanisms to escape antitumor immunity during disease progression and under treatment. Among them, genetic alterations and impaired immune function induced by chemotherapy might affect the efficacy of subsequent immune checkpoint blockade therapy upon relapse. Our observation may contribute to understanding the complex signaling network within cHL TME and may help to explore new immunotherapy approaches for resistant cases.

A main limitation of our study was the limited number of patients, preventing deeper exploration of tumor niche specificities within cHL subgroups. In particular, the effect of EBV status (n = 9 EBV positive samples, including 5 nodular sclerosis and 4 mixed-cellularity subtypes) on immune signatures across histological patterns could be analyzed by including additional EBV+ cases. Moreover, our approach was based on targeted gene expression profiling and performed on bulk samples, and therefore could not reliably determine the hallmarks of deconvoluted lymphocytes. Our cohort also lacked enough material for multiplex immunohistochemical or immunofluorescent staining, which would have been relevant for assessing the distribution of immune TME components according to histological subtype. Finally, future studies using single-cell RNA sequencing and spatial transcriptomic methods that allow the characterization of various cellular subsets and their localization at the single-cell level would better unravel cHL tumor heterogeneity and identify cellular mechanisms involved in relapse.

## 5. Conclusions

Our results highlight immune microenvironment variations in cHL during its clinical course from primary diagnosis to relapse, depending on histological subtype. Based on 586 immune-related genes GEP, we identified a 19-gene prognostic signature associated with high risk of relapse in cHL patients. This signature appeared to be mainly driven by nodular sclerosis cHL and to be irrelevant for the mixed-cellularity subtype. We also showed that the immune profile switches at relapse in the nodular sclerosis subtype, but not in the mixed-cellularity subtype. In the era of personalized medicine, our results suggest that different histological subtypes of cHL should be considered in clinical studies evaluating prognostic or predictive biomarkers, as well as in clinical trials assessing new immunomodulatory therapeutic strategies.

## Figures and Tables

**Table 1:** Clinical and histological features of 42 classical Hodgkin lymphoma patients.

	All cHL Patients N = 42	r/r cHL Patients n = 30	non-r/r cHL Patients n = 12
**Age, median (range), year**	30.5 (15–83)	30 (15–83)	31 (23–58)
**Histological subtypes, *n* (%)**			
Nodular sclerosis	24 (57.1)	17 (56.6)	7 (58.3)
Mixed-cellularity	15 (35.7)	11 (36.7)	4 (33.4)
Lymphocyte-rich	2 (4.8)	2 (6.7)	0
Lymphocyte-depleted	1 (2.4)	0	1 (8.3)
**EBV status, *n* (%)**			
EBV positive	8 (19.0)	5 (16.7)	3 (25.0)
EBV negative Missing	29 (69.0) 5 (12.0)	22 (73.3) 3 (10.0)	7 (58.3) 2 (16.7)
**Ann Arbor Stage, *n* (%)**			
I-II	9/41 (22)	9/29 (31)	0
III-IV	32/41 (78)	20/29 (69)	12 (100)
**Treatment, *n* (%)**			
BEACOPP	11 (26.2)	6 (20.0)	5 (41.7)
ABVD	24 (57.1)	17 (56.6)	7 (58.3)
Other chemotherapy *	5 (11.9)	5 (16.7)	0
Missing	2 (4.8)	2 (6.7)	0
**Treatment response, *n* (%)**			
Complete remission	35 (83.3)	23 (76.7)	12 (100)
Progression	6 (14.3)	6 (20)	0
Missing	1 (2.4)	1 (3.3)	0
**Death, *n* (%)**	8 (19)	8 (26.7)	0

Abbreviations: ABVD, doxorubicin, bleomycin, vinblastine, dacarbazine; BEACOPP, bleomycin, etoposide, doxorubicin, cyclophosphamide, vincristine, procarbazine, and prednisone; cHL, clas-sical Hodgkin lymphoma; EBV, Epstein-Barr virus; r/r, relapse/refractory. * Other chemotherapies included OEPA/COPDAC (vincristine, etoposide, prednisone, doxorubicin/cyclophosphamide, vincristine, prednisone, dacarbazine); COPP/ABV (cyclophosphamide, oncovin, procarbazine and prednisolone/adriamycine, bleomycine, and vincristine) and R-CHOP (rituximab, cyclophospha-mide, doxorubicin, vincristine, and prednisone).

## Data Availability

The data discussed in this publication have been deposited in NCBI’s Gene Expression Omnibus (Edgar et al., 2002) and are accessible through GEO Series accession number GSE212902 (https://www.ncbi.nlm.nih.gov/geo/query/acc.cgi?acc=GSE212902, accessed on 10 October 2022).

## Supplementary Materials

The following supporting information can be downloaded at: https://www.mdpi.com/article/10.3390/cancers14194893/s1, Table S1: List of genes included in the Immunology V2 panel, Table S2. Differentially expressed genes between paired diagnostic and relapse samples of classical Hodgkin lymphoma patients. Table S3. Differentially expressed genes at relapse compared to paired diagnostic samples of nodular sclerosis classical Hodgkin lymphoma patients. Figure S1. Unsupervised sample mapping is not driven by outcomes. Figure S2. Differential gene expression analysis identifies specific immune signatures at diagnosis according to the mixed-cellularity and the nodular sclerosis subtypes, all outcomes combined. Figure S3. Correlation matrix showing the interrelationship between the gene expression levels of the immune prognostic signature and the Ann Arbor stage. Figure S4. Correlation matrix showing the interrelationship among mixed-cellularity cHL samples.
