# Supplementary material for: Histological Subtypes Drive Distinct Prognostic Immune Signatures in Classical Hodgkin Lymphoma
Source: Cancers (Basel). 2022 Oct 6;14(19):4893. doi: 10.3390/cancers14194893 (PMC9563296; doi:10.3390/cancers14194893)
Supplement: Supplementary file 1 [file cancers-14-04893-s001.zip › cancers-1878252-supplementary.pdf]

Supplementary material: Histological subtypes drive distinct prognostic immune signatures in classical Hodgkin lymphoma.

Claire Lamaison, Juliette Ferrant, Pauline Gravelle, Alexandra Traverse-Glehen, Hervé Ghesquières, Marie Tosolini, Cédric Rossi, Loic Ysebaert, Pierre Brousset, Camille Laurent and Charlotte Syrykh

Table S1. List of immune related genes included in the Immunology V2 panel, with Panel Plus.

ABCB1	IRF1	IFIH1	CCBP2	EGR1
ABL1	IRF3	IFIT2	CCRL1	EGR2
ADA	IRF4	IFITM1	CD97	ENTPD1
AHR	IRF5	IFNA2	IL28A	EOMES
AICDA	IRF7	IFNAR1	IL29	ETS1
AIRE	IRF8	IFNAR2	IL8	FADD
APP	IRGM	IFNB1	KIR_Inhibiting_Subgroup_2	FAS
ARG1	ITGA2B	IFNG	C4A/B	FCAR
ARG2	ITGA4	IFNGR1	CTLA4-TM	FCER1A
ARHGDIB	ITGA5	IGF2R	CD45R0	FCER1G
ATG10	ITGA6	IKBKAP	FCGR3A/B	FCGR2A
ATG12	ITGAE	IKBKB	CTLA4_all	FCGR2B
ATG16L1	ITGAL	IKBKE	FCGR2A/C	MAPK1
ATG5	ITGAM	IKBKG	KIR_Activating_Subgroup_2	MAPK11
ATG7	ITGAX	IKZF1	KIR_Inhibiting_Subgroup_1	MAPK14
ATM	ITGB1	IKZF2	IFNA1/13	MAPKAPK2
B2M	ITGB2	IKZF3	KIR_Activating_Subgroup_1	MARCO
B3GAT1	ITLN1	IL10	IL28A/B	MASP1
BATF	ITLN2	IL10RA	PTPRC_all	MASP2
BATF3	JAK1	IL11RA	CD45RB	MBL2
BAX	JAK2	IL12A	sCTLA4	MBP
BCAP31	JAK3	IL12B	CD45RA	MCL1
BCL10	KCNJ2	IL12RB1	FCGR1A/B	MIF
BCL2	KIR3DL1	IL13	CD247	MME
BCL2L11	KIR3DL2	IL13RA1	CD27	MR1
BCL3	KIR3DL3	IL15	CD274	MRC1
BCL6	KIT	IL16	CD276	MS4A1
BID	KLRAP1	IL17A	CD28	MSR1
BLNK	KLRB1	IL17B	CD34	MUC1
BST1	KLRC1	IL17F	CD36	MX1
BST2	KLRC2	IL18	CD3D	MYD88
BTK	KLRC3	IL18R1	CD3E	NCAM1
BTLA	KLRC4	IL18RAP	CD3EAP	NCF4
C14orf166	KLRD1	IL19	CD4	NCR1
C1QA	KLRF1	IL1A	CD40	NFATC1
C1QB	KLRF2	IL1B	CD40LG	NFATC2
C1QBP	KLRG1	IL1R1	CD44	NFATC3
C1R	KLRG2	IL1R2	CD46	NFIL3
C1S	KLRK1	IL1RAP	CD48	NFKB1
C2	LAG3	IL1RL1	CD5	NFKB2
C3	LAIR1	IL1RL2	CD53	NFKBIA
C4BPA	LAMP3	IL1RN	CD55	NFKBIZ
C5	LCK	IL2	CD58	NLRP3
C6	LCP2	IL20	CD59	NOD1
C7	LEF1	IL21	CD6	NOD2
C8A	LGALS1	IL21R	CD7	NOS2

C8B	LGALS3	IL22	CD70	NOTCH1
C8G	LIF	IL22RA2	CD74	NOTCH2
C9	LILRA1	STAT6	CD79A	NT5E
CAMP	LILRA2	SYK	CD79B	PAX5
CARD9	LILRA3	TAGAP	CD80	PDCD1
CASP1	LILRA4	TAL1	CD81	PDCD1LG2
CASP10	LILRA5	TAP1	CD82	PDCD2
CASP2	LILRA6	TAP2	CD83	PDGFB
CASP3	LILRB1	TAPBP	CD86	PDGFRB
CASP8	LILRB2	TBK1	CD8A	PECAM1
CCL11	LILRB3	TBX21	CD8B	PIGR
CCL13	LILRB4	TCF4	CD9	PLA2G2A
CCL15	LILRB5	TCF7	CD96	PLA2G2E
CCL16	LITAF	TFRC	CD99	PLAU
CCL18	LTA	TGFB1	CDH5	PLAUR
CCL19	LTB4R	TGFBI	CDKN1A	PML
CCL2	LTB4R2	TGFBR1	CEACAM1	POU2F2
CCL20	LTBR	TGFBR2	CEACAM6	PPARG
CCL22	LTF	THY1	CEACAM8	PPBP
CCL23	LY96	TICAM1	CEBPB	PRDM1
CCL24	MAF	TIGIT	CFB	PRF1
CCL26	MALT1	TIMP1	CFD	PRKCD
CCL3	MAP4K1	TIRAP	CFH	PSMB10
CCL4	MAP4K2	TLR1	CFI	PSMB5
CCL5	MAP4K4	TLR2	CFP	PSMB7
CCL7	FCGRT	TLR3	CHUK	PSMB8
CCL8	FKBP5	TLR4	CIITA	PSMB9
CCND3	FN1	TLR5	CISH	PSMC2
CCR1	FOXP3	TLR7	CLEC4A	PSMD7
CCR10	FYN	TLR8	CLEC4E	PTAFR
CCR2	GATA3	TLR9	CLEC5A	PTGER4
CCR5	GBP1	TMEM173	CLEC6A	PTGS2
CCR6	GBP5	TNF	CLEC7A	PTK2
CCR7	GDF15	TNFAIP3	CLU	PTPN2
CCR8	GFI1	TNFAIP6	CMKLR1	PTPN22
CCRL2	GNLY	TNFRSF10C	CR1	PTPN6
CD14	GP1BB	TNFRSF11A	CR2	PVR
CD160	GPI	TNFRSF13B	CRADD	PYCARD
CD163	GPR183	TNFRSF13C	CSF1	RAF1
CD164	GZMA	TNFRSF14	CSF1R	RAG1
CD19	GZMB	TNFRSF17	CSF2	RAG2
CD1A	GZMK	TNFRSF1B	CSF2RB	RARRES3
CD1D	HAMP	TNFRSF4	CSF3R	RELA
CD2	HAVCR2	TNFRSF8	CTNNB1	RELB
CD209	HFE	TNFRSF9	CTSC	RORC
CD22	HGF	TNFSF10	CTSG	RUNX1
CD24	HLA-A	TNFSF11	CTSS	S100A8
CD244	HLA-B	TNFSF12	CUL9	S100A9
IL23A	HLA-C	TNFSF13B	CX3CL1	S1PR1
IL23R	HLA-DMA	TNFSF15	CX3CR1	SELE
IL26	HLA-DMB	TNFSF4	CXCL1	SELL
IL27	HLA-DOB	TNFSF8	CXCL10	SELPLG
IL2RA	HLA-DPA1	TOLLIP	CXCL11	SERPING1
IL2RB	HLA-DPB1	TP53	CXCL12	SH2D1A
IL2RG	HLA-DQA1	TRAF1	CXCL13	SIGIRR
IL3	HLA-DQB1	TRAF2	CXCL2	SKI

IL32	HLA-DRA	TRAF3	CXCL9	SLAMF1
IL4	HLA-DRB1	TRAF4	CXCR1	SLAMF6
IL4R	HLA-DRB3	TRAF5	CXCR2	SLAMF7
IL5	HRAS	TRAF6	CXCR3	SLC2A1
IL6	ICAM1	TYK2	CXCR4	SMAD3
IL6R	ICAM2	UBE2L3	CXCR6	SMAD5
IL6ST	ICAM3	VCAM1	CYBB	SOCS1
IL7	ICAM4	VEGFA	DEFB1	SOCS3
IL7R	ICAM5	VTN	DEFB103A	SPP1
IL9	ICOS	XBP1	DEFB103B	SRC
ILF3	ICOSLG	XCL1	DEFB4A	STAT1
IRAK1	IDO1	XCR1	DPP4	STAT2
IRAK2	IDO2	ZAP70	DUSP4	STAT3
IRAK3	IFI16	ZBTB16	EBI3	STAT4
IRAK4	IFI35	ZEB1	EDNRB	STAT5A
				STAT5B

Table S2. Differentially expressed genes between paired diagnostic and relapse samples of classical Hodgkin lymphoma patients.

Global diagnosis signature				
**Genes**	**Fold.Change**	**Log.Fold.Change**	**p.Value**	p.Adj
IRAK3	-4,31084	-2,10797	4,69E-07	0,0000361
KLRG2	-3,99687	-1,99887	0,002384	0,009989
C5	-3,96585	-1,98763	0,000271	0,002199
TNFSF15	-3,76918	-1,91425	0,0000151	0,000295
IL15	-3,59437	-1,84574	0,0000603	0,000845
LILRA3	-3,33852	-1,73921	0,00076	0,004499
LILRA5	-3,22212	-1,68801	0,0000278	0,000503
FCGR2A/C	-3,14459	-1,65287	0,000354	0,002598
HGF	-2,98912	-1,57972	0,001558	0,007624
FCGR3A/B	-2,87438	-1,52325	0,00127	0,006744
TAL1	-2,80272	-1,48683	0,001713	0,007873
TNFRSF17	-2,78887	-1,47968	0,000659	0,004319
NCAM1	-2,78286	-1,47657	0,002771	0,010747
XCR1	-2,74697	-1,45784	0,000538	0,003834
NFATC3	-2,74151	-1,45497	0,001538	0,007624
TRAF6	-2,59059	-1,37328	0,000165	0,001539
GZMB	-2,55574	-1,35374	0,001979	0,008833
PTGS2	-2,55556	-1,35364	0,00481	0,015929
LILRB1	-2,54601	-1,34824	0,008265	0,023571
LTA	-2,46882	-1,30382	0,000147	0,001539
C14orf166	-2,44665	-1,29081	0,000155	0,001539
CD163	-2,39283	-1,25872	0,004302	0,014561
CD274	-2,314	-1,21039	0,000726	0,004499
KCNJ2	-2,28351	-1,19125	0,004229	0,014472
PSMB7	-2,25538	-1,17337	0,0000706	0,000906
CD1A	-2,23991	-1,16344	0,009853	0,026161
C6	-2,23485	-1,16018	0,008411	0,023766
ICOS	-2,21352	-1,14634	0,002457	0,009989
PSMB5	-2,21335	-1,14623	0,005882	0,018873
TNFSF4	-2,20866	-1,14317	0,006186	0,019643
TLR1	-2,16715	-1,1158	0,002633	0,010397
TNFSF13B	-2,16174	-1,11219	0,011531	0,029597
NOD2	-2,15453	-1,10737	0,001526	0,007624
LILRB2	-2,1477	-1,10279	0,001507	0,007624

CLEC4E	-2,1319	-1,09214	0,008218	0,023571
MAPK11	-2,08289	-1,05859	0,001135	0,006245
CASP1	-2,07393	-1,05237	0,002873	0,010923
NOD1	-2,06394	-1,0454	0,009071	0,024945
ITGAM	-2,03689	-1,02637	0,003519	0,013057
IL21	-2,03094	-1,02215	0,009398	0,02539
TLR4	-1,95145	-0,96455	0,018149	0,041715
TICAM1	-1,95019	-0,963611	0,009602	0,025717
IL18R1	-1,93361	-0,951296	0,020067	0,045113
IL1R2	-1,9309	-0,949274	0,012434	0,0312
LILRA6	-1,92718	-0,946491	0,00649	0,019989
CD28	-1,9257	-0,945386	0,005597	0,018146
IL1RAP	-1,91036	-0,933842	0,007243	0,021872
CR1	-1,88689	-0,91601	0,002014	0,008864
MYD88	-1,8858	-0,915179	0,000758	0,004499
IL7R	-1,84346	-0,88242	0,019891	0,045048
ATG10	-1,75647	-0,812682	0,008796	0,024406
CD48	-1,71236	-0,775986	0,009369	0,02539
SLAMF7	-1,62172	-0,697527	0,018004	0,041694
TLR2	-1,5894	-0,668481	0,012828	0,031357
STAT4	-1,58875	-0,667892	0,010343	0,027221

Global relapse signature

**Genes**	**Fold.Change**	**Log.Fold.Change**	**p.Value**	p.Adj
CXCR3	1,65214	0,724334	0,017763	0,041663
LITAF	1,85276	0,889676	0,013421	0,032044
TRAF1	1,86585	0,899833	0,011768	0,029954
CLEC4A	1,88134	0,911762	0,012628	0,031317
CCL5	1,90041	0,926307	0,008242	0,023571
CD3E	1,91234	0,935338	0,013295	0,031992
ARHGDIB	1,94414	0,959132	0,004659	0,015599
STAT3	1,94735	0,961515	0,002314	0,009897
CD8A	1,95426	0,966626	0,000213	0,00187
STAT6	2,05777	1,04108	0,000036	0,000555
PSMB9	2,07189	1,05095	0,000297	0,002285
HLA-DMA	2,08414	1,05945	0,001132	0,006245
ZAP70	2,09087	1,0641	0,019377	0,044208
CD82	2,09324	1,06574	0,01313	0,031844
B2M	2,10466	1,07359	0,008703	0,024368
BST2	2,14044	1,09791	0,007654	0,022667
IRF8	2,16066	1,11147	0,007573	0,022646
CYBB	2,16424	1,11386	0,000845	0,004822
IFI16	2,1874	1,12922	0,002465	0,009989
ITGAL	2,19082	1,13147	0,003619	0,01327
PTPRC_all	2,20745	1,14238	0,00633	0,019694
SOCS3	2,27609	1,18656	0,004131	0,014425
FCGRT	2,39144	1,25788	0,00262	0,010397
CCL19	2,44056	1,28721	0,005347	0,01752
ITGB2	2,45668	1,29671	0,00156	0,007624
CD34	2,47997	1,31032	0,01246	0,0312
TNFRSF8	2,48198	1,31149	0,004136	0,014425
CD3D	2,48437	1,31288	0,001693	0,007873
HLA-DRA	2,53905	1,34429	0,011312	0,029277
TGFB1	2,55319	1,3523	0,001635	0,007869
C7	2,55693	1,35441	0,010429	0,027221
NCF4	2,60684	1,3823	0,002791	0,010747

IRF4	2,69322	1,42933	0,000253	0,00211
CD4	2,77783	1,47396	0,001691	0,007873
TAPBP	2,78535	1,47786	0,000163	0,001539
IFI35	2,8051	1,48805	0,001234	0,00667
IKZF3	2,86185	1,51695	0,000796	0,004628
GBP5	2,86642	1,51925	0,000631	0,004224
RELA	2,91311	1,54256	0,000615	0,00421
LAG3	2,91727	1,54462	0,003908	0,013995
EBI3	2,9868	1,5786	0,001837	0,008323
C1QBP	2,99621	1,58314	0,000548	0,003834
CD81	2,99638	1,58322	0,007225	0,021872
CD99	2,99719	1,58361	0,0000932	0,001104
LTBR	3,00083	1,58536	0,000759	0,004499
JAK3	3,07134	1,61887	0,00222	0,009631
CD79B	3,09337	1,62918	0,000288	0,002272
IKBKAP	3,15325	1,65684	0,000354	0,002598
BCL3	3,16944	1,66423	0,000148	0,001539
PSMB10	3,32771	1,73453	0,000238	0,002037
BATF	3,49449	1,80508	0,000164	0,001539
CD74	3,49858	1,80677	0,0000112	0,000295
CFD	3,59066	1,84425	0,000203	0,001835
HLA-DRB1	3,59564	1,84625	0,004168	0,014425
IL32	3,65414	1,86953	0,0000641	0,000859
PML	3,70949	1,89122	0,0000319	0,000522
TNFAIP3	4,00211	2,00076	0,0000119	0,000295
CD79A	4,22438	2,07874	0,000105	0,0012
BCAP31	4,43198	2,14795	0,01271	0,031317
NFKBIA	4,50176	2,17049	0,0000153	0,000295
FCER1G	4,57271	2,19305	0,0000526	0,000771
IRF3	4,96058	2,31051	0,0000322	0,000522
CD7	4,9715	2,31368	0,017246	0,04086
LY96	4,98962	2,31893	0,0000133	0,000295
PTPN2	5,50324	2,46028	0,00000131	0,00007
HLA-C	5,56322	2,47592	0,00000156	0,00007
CD14	5,70944	2,51335	0,00000451	0,000139
APP	5,72518	2,51732	0,000015	0,000295
CXCL13	5,77544	2,52993	0,0000779	0,00096
HLA-B	6,27715	2,65011	3,52E-08	0,0000108
FYN	6,51006	2,70267	0,003172	0,011915
TNFRSF1B	6,8403	2,77406	0,002404	0,009989
TGFBI	7,01873	2,81121	0,0000031	0,000114
TNFSF12	7,75706	2,95551	0,00000159	0,00007
CD45R0	8,06039	3,01085	0,006291	0,019694
TMEM173	8,32846	3,05805	3,33E-07	0,0000342
MS4A1	8,42023	3,07386	0,00000334	0,000114
HLA-A	8,73053	3,12607	1,15E-07	0,0000176
LGALS1	10,6086	3,40716	0,000733	0,004499

Table S3. Differentially expressed genes at relapse compared to paired diagnostic samples of nodular sclerosis classical Hodgkin lymphoma patients.

**Up and down regulated genes at relapse in the nodular sclerosis subtype.**				
**genes**	**Fold.Change**	**Log.Fold.Change**	**p.Value**	p.Adj
TNFSF15	-9,51761	-3,2506	0,00000442	0,000136
IRAK3	-7,53423	-2,91346	0,00000561	0,000157

LILRB2	-6,02153	-2,59013	0,00000166	0,0000598
C5	-6,00548	-2,58628	0,004454	0,017142
LILRA5	-5,82105	-2,54128	0,0000674	0,000988
TAL1	-5,62175	-2,49102	0,001574	0,008505
IL1R2	-5,50228	-2,46003	0,0000713	0,000998
IL15	-5,4402	-2,44366	0,001712	0,008939
HGF	-5,29382	-2,40431	0,003355	0,014353
PTGS2	-5,20605	-2,38019	0,001011	0,006104
NCAM1	-5,06756	-2,34129	0,006633	0,023481
LTA	-4,7406	-2,24507	0,0000127	0,000245
CD1A	-4,66795	-2,22279	0,002065	0,010098
FCGR2A/C	-4,20277	-2,07134	0,002656	0,012211
GZMB	-4,18895	-2,06659	0,002452	0,011444
KCNJ2	-4,15783	-2,05583	0,001413	0,007773
TNFRSF17	-4,02405	-2,00865	0,00379	0,015484
TNFSF4	-3,99128	-1,99685	0,004133	0,01632
C6	-3,98633	-1,99506	0,005777	0,021181
FCGR3A/B	-3,98602	-1,99495	0,00609	0,022068
ABCB1	-3,93136	-1,97503	0,004748	0,017621
CD274	-3,7341	-1,90076	0,000688	0,004712
IL1RAP	-3,71263	-1,89244	0,00098	0,006104
TRAF6	-3,56435	-1,83364	0,001264	0,007209
CD163	-3,46227	-1,79172	0,010075	0,031666
XCR1	-3,46208	-1,79164	0,013124	0,037362
C14orf166	-3,29816	-1,72166	0,000913	0,00598
MME	-3,28388	-1,7154	0,016376	0,043859
IL18R1	-3,20095	-1,6785	0,008537	0,028226
PSMB7	-3,0902	-1,6277	0,00029	0,002627
MAPK11	-3,07627	-1,62118	0,001841	0,009359
CCR5	-2,96286	-1,56699	0,008663	0,028226
GZMA	-2,93628	-1,55399	0,004508	0,017142
CD276	-2,93618	-1,55394	0,011137	0,034093
CASP3	-2,91851	-1,54523	0,006563	0,023481
TICAM1	-2,85255	-1,51225	0,011751	0,034519
ICOS	-2,82431	-1,4979	0,01188	0,034519
MALT1	-2,66469	-1,41397	0,018631	0,04863
SLAMF7	-2,53336	-1,34105	0,003057	0,013369
CASP1	-2,45973	-1,2985	0,013614	0,038118
TLR1	-2,44871	-1,29202	0,018338	0,048274
NOD2	-2,40127	-1,2638	0,01634	0,043859
MYD88	-2,22026	-1,15073	0,003617	0,015056
CHUK	-1,94858	-0,962426	0,000951	0,006104
MAPK14	1,88933	0,917874	0,008706	0,028226
PSMB9	2,23803	1,16223	0,010749	0,033441
STAT6	2,45654	1,29663	0,000906	0,00598
IFI16	2,54561	1,34801	0,016745	0,044462
STAT3	2,63051	1,39534	0,002811	0,012734
LITAF	2,70079	1,43338	0,008274	0,027699
CRADD	2,78947	1,47999	0,011492	0,034504
HLA-DMA	2,80761	1,48934	0,002242	0,010788
TGFBR2	2,81565	1,49347	0,01118	0,034093
CD3D	2,89933	1,53572	0,015962	0,043506
IRF8	3,0282	1,59846	0,014481	0,039822
CD3E	3,06522	1,61599	0,004584	0,017219
PTPRC_all	3,18358	1,67065	0,011539	0,034504
CLEC4A	3,21188	1,68342	0,002296	0,010879

ZAP70	3,38257	1,75812	0,013222	0,037362
CD82	3,38295	1,75828	0,009337	0,02965
CCL7	3,43517	1,78038	0,01179	0,034519
C1QBP	3,44891	1,78614	0,008019	0,027142
ITGAL	3,46175	1,7915	0,003849	0,015484
TRAF1	3,47902	1,79868	0,001938	0,00963
CYBB	3,52289	1,81676	0,00109	0,006435
FCGRT	3,59236	1,84493	0,003082	0,013369
IKZF3	3,62103	1,8564	0,00777	0,02659
B2M	3,97711	1,99172	0,002938	0,013114
CCL19	4,01019	2,00367	0,003871	0,015484
NCF4	4,01403	2,00505	0,004278	0,01668
STAT1	4,12373	2,04395	0,000574	0,004113
IRF4	4,31862	2,11057	0,000338	0,002827
TAPBP	4,32629	2,11313	0,00034	0,002827
TGFB1	4,34104	2,11804	0,001342	0,007513
CFD	4,39103	2,13456	0,006989	0,024186
SOCS3	4,45035	2,15392	0,001107	0,006435
PSMB10	4,47833	2,16296	0,001693	0,008939
CD99	4,80918	2,26579	0,000136	0,001513
MIF	4,93226	2,30225	0,009338	0,02965
IFI35	5,24932	2,39213	0,001854	0,009359
TNFRSF4	5,34156	2,41726	0,003443	0,014525
TNFAIP3	5,41173	2,43609	0,00042	0,003384
PML	5,62526	2,49192	0,000136	0,001513
RELA	5,80055	2,53619	0,000642	0,004496
NOTCH2	5,84714	2,54773	0,006747	0,023615
EBI3	5,98512	2,58138	0,001006	0,006104
LTBR	6,00815	2,58692	0,000319	0,002811
TNFRSF8	6,02024	2,58982	0,000429	0,003384
CD4	6,12237	2,61409	0,000253	0,002359
GBP5	6,23098	2,63946	0,00011	0,001357
CD79B	6,37524	2,67248	0,00021	0,00202
ITGB2	6,41967	2,6825	0,0000994	0,001275
CD74	6,45197	2,68974	0,00000737	0,000159
NFKBIA	7,07956	2,82366	0,00045	0,003384
BATF	7,09258	2,82631	0,000142	0,001513
BCAP31	7,3403	2,87584	0,000169	0,001674
BCL3	7,82402	2,96791	0,00000654	0,000158
HLA-DRA	8,01321	3,00238	0,000445	0,003384
JAK3	8,30834	3,05456	0,000552	0,00405
IKBKAP	8,6187	3,10747	0,0000341	0,000554
CD79A	9,08345	3,18324	0,000155	0,001594
CD14	9,95506	3,31543	0,0000403	0,000621
HLA-A	10,1512	3,34358	0,013215	0,037362
FCER1G	10,3586	3,37276	0,0000822	0,001101
LY96	11,4447	3,51661	0,0000262	0,000448
APP	12,0658	3,59285	0,00014	0,001513
HLA-B	12,1013	3,59709	3,19E-07	0,000019
MS4A1	12,929	3,69254	0,014332	0,039767
HLA-C	13,652	3,77104	4,27E-07	0,000019
CD7	14,0721	3,81477	0,0000146	0,000264
PTPN2	16,6213	4,05496	1,06E-07	0,0000163
TNFSF12	16,7178	4,06331	0,00000776	0,000159
IRF3	18,9141	4,24139	0,00000175	0,0000598
FYN	20,0892	4,32835	1,05E-07	0,0000163

TGFBI	21,5955	4,43266	0,00000666	0,000158
TNFRSF1B	24,0544	4,58823	4,32E-07	0,000019
TMEM173	25,5895	4,67748	3,23E-07	0,000019
LGALS1	36,1913	5,17757	2,68E-07	0,000019

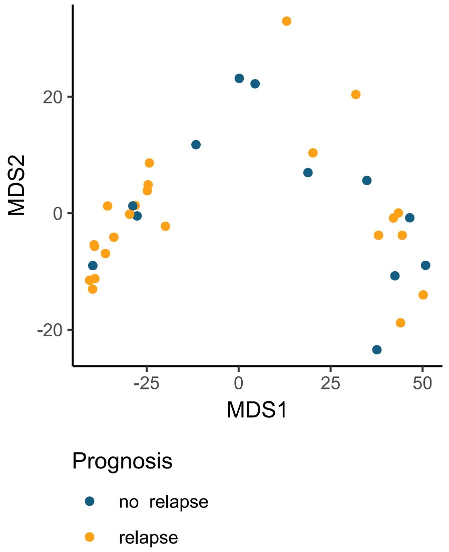

Figure S1. Unsupervised samples mapping is not driven by outcomes. Multidimensional scaling (MDS) plots of diagnostic biopsies (n=35) based on the Immunology V2 Nanostring panel gene expression.

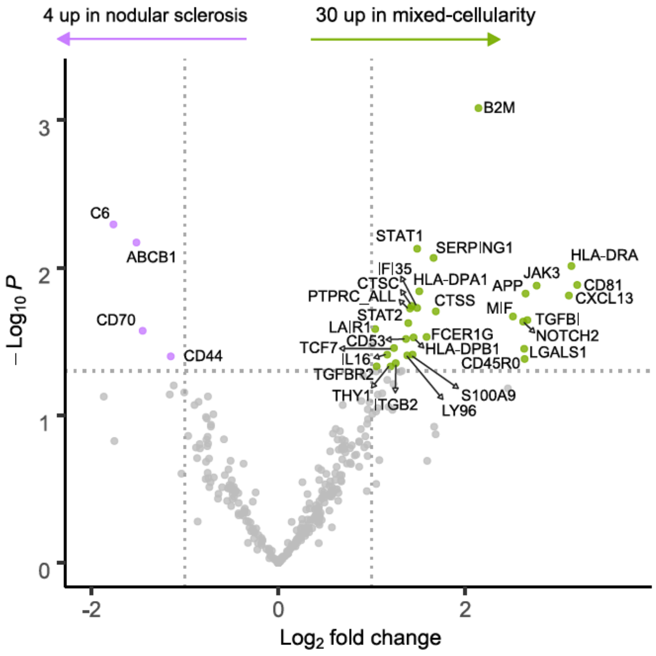

Figure S2. Differential gene expression analysis identifies specific immune signatures at diagnosis according to the mixed-cellularity and the nodular sclerosis subtypes, all outcomes combined. Volcano plot displaying differentially expressed genes at diagnosis between the nodular sclerosis and the mixed-cellularity subtypes. Genes significantly deregulated ($p < 0.05$ and $|\text{fold change}| > 2$) are highlighted in purple (upregulated in the nodular sclerosis subtype, n=4) or green (upregulated in the mixed-cellularity subtype, n=30).

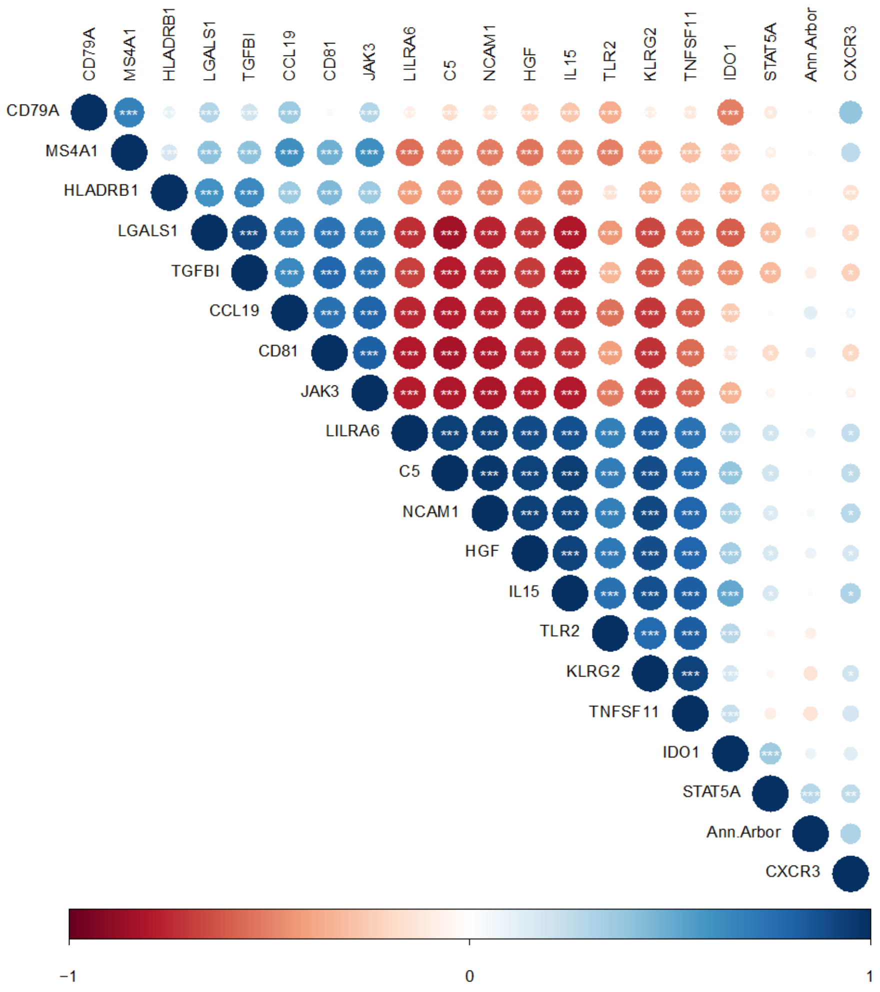

Figure S3. Correlation matrix showing the interrelationship between the gene expression levels of the immune prognostic signature and the Ann Arbor stage. Spearman correlation performed. Samples are ordered by hierarchical clustering, negative correlations are displayed in red and positive correlations in blue. *** p <0.001, **p< 0.01, *p< 0.05

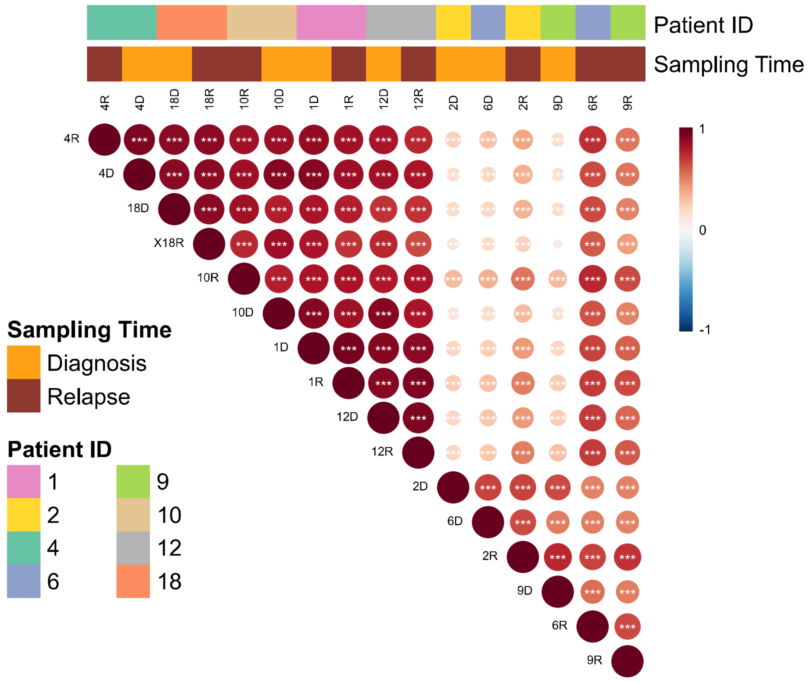

Figure S4. Correlation matrix showing the interrelationship among mixed-cellularity cHL samples. Pearson correlation performed on Immunology V2 Nanostring panel gene expression. Samples are ordered by hierarchical clustering, positive correlations are displayed in red and negative correlations in blue. *** p <0.001, **p< 0.01, *p< 0.05
